# Influence of the renewal of removable dentures on oral health related quality of life

**DOI:** 10.1186/s40064-016-3699-7

**Published:** 2016-11-28

**Authors:** Guillaume Bonnet, Cindy Batisse, Jean W. Segyo, Jean-Luc Veyrune, Emmanuel Nicolas, Marion Bessadet

**Affiliations:** 1 Clermont Université, Université d’Auvergne, EA4847, CROC, BP 10448, 63000 Clermont-Ferrand, France; 2 CHU Clermont-Ferrand, Service d’Odontologie, 63003 Clermont-Ferrand, France

## Abstract

**Background:**

The renewal of removable dentures is often suggested to denture wearers subject to discomfort. However, the impact of this rehabilitation on patients’ oral health related quality of life and their removable dentures related satisfaction is still unknown. This study was aimed at assessing these patient-centered outcomes and the potential impact of different factors.

**Methods:**

A cohort of 116 patients in need of removable dental prostheses rehabilitation was recruited at a dental hospital over a period of 1 year. The subjects were separated into two groups according to their prosthesis experience (group in need of removable dentures renewal/group needing an removable dentures for the first time). Subjects were asked to answer the “Geriatric Oral Health Assessment Index” (GOHAI) and the “McGill Denture Satisfaction Instrument” before and after a prosthesis integration period (9–12 weeks).

**Results:**

GOHAI scores were slightly higher for patients with removable dentures renewal (from 40.6 ± 10.3 to 47.1 ± 10.0, p < 0.001), independently of the type of prosthetic rehabilitation. However, the scores of the GOHAI functional field did not change. Subjects with no removable dentures experience presented an increase in their functional GOHAI score (p < 0.001). Regarding patient removable dentures related satisfaction, only the “Esthetic” (p < 0.001), “Chewing efficiency” (p < 0.001) and “Oral condition” (p < 0.01) items increased after prosthesis renewal.

**Conclusions:**

This study showed that renewing removable dentures only moderately improved the oral health related quality of life and removable dentures related satisfaction of patients, regardless of age, gender or type of rehabilitation. Other tasks are necessary such as the analysis of physiological parameters and qualitative research on patient’s expectations.

## Background

Worldwide, the frequency of edentulism over 65 years of age fluctuates considerably between countries (26% in the USA, 19% in Italy, and 46% in the United Kingdom, to name but a few) (Petersen and Yamamoto 2005). In France, the prevalence of edentulism continues to increase in the population, from 16.3% in 1995 to 23.8% in 2004 (Haute Autorité de Santé 2006), and it is a phenomenon that can be partially explained by population ageing. No data is available on the proportion of partially edentate persons, which would further increase the previous percentage if taken into account. Prosthetic treatment with removable dentures (RD) represents one of the therapeutic treatments available against tooth loss, and it is also that used most frequently. Esthetics and oral functions such as phonation and mastication should be restored (Roumanas 2009) following RD placement and patients should recover “good” oral health. The definition of oral health is not limited to the absence of pathology. Just as the World Health Organization’s states that “health is a state of complete physical, mental and social well-being and not merely the absence of disease or infirmity” (World Health Organisation 1948), oral health is defined as a “state of being free from mouth and facial pain, oral and throat cancer, oral infection and sores, periodontal (gum) disease, tooth decay, tooth loss, and other diseases and disorders that limit an individual’s capacity for biting, chewing, smiling and speaking, and their psychosocial well-being” (World Health Organisation 2012). In the case of tooth loss, rehabilitation of oral health by wearing an RD does not allow patients to return to a state of oral health as defined above. Indeed, the literature showed that oral health related quality of life (OHRQoL) measured using the “Geriatric Oral Health Assessment Index” (GOHAI) (Atchison et al. 1998) remained deteriorated in the presence of RD (Locker and Miller 1994; McGrath and Bedi 2001; John et al. 2004). Similar results were shown using the shortened version of Oral Health Impact Profile (OHIP-14) and OHIP-edent questionnaires (Pistorius et al. 2013; Peršić and Čelebić 2015; Yen et al. 2015). RD renewal is often suggested to solve patients’ discomfort and grievances, and/or denture wear. However, few data are available on the impact of RD renewal on patients’ OHRQoL and their RD-related satisfaction, and existing data mainly focus on bi-maxillary complete RD (Veyrune et al. 2005). Moreover, in France, the evaluation of practice has recently become a key objective within university hospitals and private practices, in order to improve the quality of health care. In this context, the evaluation of RD renewal would undoubtedly contribute to improving the quality of oral health care. Such evaluation remains difficult, especially for prosthetics rehabilitation, because of the wide variety of existing processes. It has therefore been decided to focus on patient-centered outcomes, as done by Donabedian (2005).

Within this scope, this study aimed at assessing whether the OHRQoL and prosthesis-related satisfaction in patients that have undergone RD renewal was improved. The impact of sociodemographic factors such as age and gender, and the type of rehabilitation, were also evaluated.

## Methods

### Participants’ characteristics

A total of 127 subjects were recruited from among patients in need of RD rehabilitation visiting the dental unit of the Clermont-Ferrand University Hospital (Rhone-Alpes-Auvergne, France) between September 2014 and September 2015. From this population, 11 subjects were excluded for the following reasons: (1) difficulty in understanding the study questionnaire (three subjects); (2) the presence of cognitive or psychological disorders (two subjects); (3) chronic orofacial pain (three subjects); and (4) refusal to participate (three subjects). In total, 116 subjects (mean age 63.7± 12.4 years ) i.e. 55 men (63.2 ± 10.8 years) and 61 women (64.1 ± 13.8 years), were included. The patients were managed by fifth and sixth year dental students under the supervision of a prosthodontic university professor (senior practitioner) in order to comply with good clinical practice. In addition, endodontic, conservative and periodontic treatments were performed before prosthesis renewal.

Sociodemographic data were recorded, including gender, age (two groups according to a 70 year-old age limit), level of education, way of life, and RD experience. Two groups of patients were determined: subjects requiring renewal of their existing RD (RD renewal), and subjects in need of RD for the first time (control). After the oral examination of all the patients, it was estimated that four types of rehabilitation were needed: (1) bi-maxillary complete RD, (2) uni-maxillary complete RD, (3) uni-maxillary partial RD, and (4) bi-maxillary partial RD. These data are presented in Table 1.

**Table 1:** Participants’ characteristics (study population) (n = 116)

Variables	Number of participants (%)
Gender	
Women	61 (52.6%)
Men	55 (47.4%)
Age (years)	
Under 70	82 (70.7%)
Over 70	34 (29.3%)
Education level	
Below high school	52 (46.8%)
High school or above	59 (53.2%)
Way of life	
Alone	44 (38.6%)
Living with another person	72 (61.4%)
RD experience	
With RD experience	79 (68.1%)
Without RD experience	37 (31.9%)
Types of prosthetic rehabilitations need	
Bi-maxillary complete RD	36 (31.0%)
Uni-maxillary complete RD	33 (28.5%)
Uni-maxillary partial RD	20 (17.2%)
Bi-maxillary partial RD	27 (23.3%)

This observational study was approved by the local ethical committee (CE-CIC GREN-09-12; IRB Number 5044). Information was given to the subjects and a consent form signed.

### Measuring instruments

#### Oral health related quality of life assessment

The OHRQoL was assessed using the French validated GOHAI version (Tubert-Jeannin et al. 2003). GOHAI comprises 12 items grouped into three fields: (1) the functional field (eating, speaking, swallowing); (2) the psychosocial field (concerns, relational discomfort, appearance); (3) the pain or discomfort field (drugs, gingival sensitivity, discomfort when chewing certain foods). The cumulative method (GOHAI-Add) was used in this study and it consists in summing the scores obtained for each of the 12 GOHAI questions. Each question is scored from 1 to 5. In this study however, subjects in need of bi-maxillary complete RD rehabilitation (with or without prosthetic experience) did not reply to the item relating to dental sensitivity to heat and cold because they were edentulous. The maximum score of 5 was therefore attributed to each subject for this item. The maximum score was 60 (20 = functional field; 25 = psychosocial field; 15 = pain or discomfort field). According to Atchison and Dolan (1990), a score of 57–60 is regarded as high and corresponds to a satisfactory OHRQoL. A score from 51 to 56 is regarded as average, and a score of 50 or less is regarded as a low score, reflecting a poor OHRQoL.

#### Patient RD-related satisfaction assessment

Patient Satisfaction related to wearing an RD was measured by the “McGill Denture Satisfaction Instrument” (MGDSI) (De Grandmont et al. 1994; Awad and Feine 1998). This questionnaire was used initially in patients with bi-maxillary complete RD to evaluate their satisfaction related to their mandibular denture. Satisfaction is assessed through nine categories of items, each containing between 1 and 8 questions, with a total of 25 questions. The categories are separated into “Ease of cleaning” “General satisfaction”, “Ability to speak” “Comfort”, “Esthetics”, “Stability”, “Chewing ability” (*is it difficult for you to eat?*), “Chewing efficiency” (*are the food particles usually well crushed before swallowing?*), and “Oral condition”. The participants answered each question using 100 mm visual analogue scales, anchored by the words “not satisfied at all” and “extremely satisfied”. Questions about chewing ability and chewing efficiency are asked for 8 distinct foods, for which only the average score was reported in this study. In our case, food illustrations, taken from the SUVIMAX iconographic method (Hercberg and Deheeger 1994), were associated with the appropriate question to facilitate comprehension. Finally, this measuring instrument was formulated for all types of rehabilitation (partial and complete RD).

### Study design

All the 116 subjects included were separated into two groups according to whether they already wore a removable denture (“with RD experience”; 79 subjects) or not (“without RD experience”; 37 subjects). During their initial visit, they all answered the GOHAI questionnaire, and only subjects with RD experience completed the MGDSI questionnaire. After rehabilitation, all the subjects had a variable follow-up period (unique to each patient), during which the patients visited the dental unit so that the dentist could perform corrective procedures as often as needed for the new RD to become an integral part of the stomatognathic system. Upon integration, an appointment was given to the subject within 9–12 weeks, as done in previous studies (Veyrune et al. 2005; Nicolas et al. 2010), for the second evaluation (according to the subjects’ availability), when they completed both the GOHAI and MGDSI questionnaires. However, during this period, several patients dropped out of the study, leading to the exclusion of their data. Therefore, on the last evaluation date following prosthesis rehabilitation the group with RD experience, named “RD Renewal” group, consisted of 43 subjects, and the group without RD experience, named “Control” group, consisted of 16 subjects. The “RD Renewal” group was subsequently divided into four categories, according to their prosthetic needs and also separated in two, according to the 70 year-old age limit (60.1 ± 6.7 and 77.4 ± 5.5 years, respectively).

This group of subjects obtained after rehabilitation (59) was comparable according to age, gender, prosthesis experience and type of rehabilitation (Chi square, non significant), as well as to the OHRQoL (t test, non significant), to the initial group of subjects (116).

### Statistical analysis

The mean scores of each component of GOHAI and MGDSI between the “Control” group and the “RD Renewal” group were compared before and after rehabilitation independently by Student t tests (α = 0.01, Bonferroni correction). For the “Renewal RD” group and for each component, differences between types of prosthetic rehabilitation were assessed by post ANOVA Student–Newman–Keuls tests (α = 0.01). The influence of gender, age group, education level and way of life was tested by a Student t test (α = 0.05).

For the “Control” group, the evolution of GOHAI was tested by a paired Student *t* test (α = 0.01). For the “Renewal RD” group, the impact of rehabilitation on GOHAI and MGDSI components was assessed by the repeated measures procedure (dependent factors: GOHAI or MGDSI, fixed factor: type of prosthetic rehabilitation α = 0.01).

## Results

### Before prosthetic rehabilitation

The mean GOHAI and MGDSI scores were reported in Table 2 for each group, according to their RD experience and type of prosthetic rehabilitation. No statistical difference was found for any of the components tested. Before rehabilitation, subjects from both the “Renewal RD” group and the “Control” group declared a poor OHRQoL (GOHAI-Add scores of 40.6 ± 10.3 and 45 ± 12.1, respectively).

**Table 2:** Comparison of GOHAI and MGDSI scores between groups of the study before rehabilitation

Variable parameters before rehabilitation	RD experience			Types of prosthetic rehabilitation (renewal RD group only)				
	Renewal RD group n = 43	Control group n = 16	Student t test	Bi-maxillary complete RD n = 17	Uni-maxillary complete RD n = 14	Uni-maxillary partial RD n = 5	Bi-maxillary partial RD n = 7	ANOVA procedure
	Mean ± SD	Mean ± SD	p value	Mean ± SD	Mean ± SD	Mean ± SD	Mean ± SD	p value
Oral health related quality of life assessment (GOHAI questionnaire)								
GOHAI-Add	40.6 ± 10.3	45 ± 12.1	0.17	36.5 ± 10.0	44.9 ± 10.9	39.2 ± 5.9	43.1 ± 10.2	0.13
Functional field	13.2 ± 4.4	13.8 ± 5.2	0.63	10.8 ± 4.8	15.2 ± 3.6	13.4 ± 2.2	14.6 ± 4.2	0.03
Psychosocial field	15.7 ± 5.1	18.6 ± 5.1	0.05	13.9 ± 5.3	17.3 ± 5.1	15.4 ± 3.4	16.7 ± 5.5	0.31
Pain or discomfort field	11.7 ± 2.5	12.6 ± 4.3	0.31	11.7 ± 2.1	11.9 ± 3.0	10.4 ± 2.5	11.9 ± 2.6	0.81
Patient’s RD-related satisfaction assessment (MGDSI questionnaire)								
Ease of cleaning	80.0 ± 26.9		NA	70.6 ± 36.2	89.0 ± 13.1	84.0 ± 27.6	94.3 ± 6.4	0.13
General satisfaction	59.7 ± 35.1		NA	51.3 ± 39.7	68.7 ± 31.8	54.8 ± 28.2	65.4 ± 29.9	0.55
Ability to speak	67.4 ± 35.6		NA	58.9 ± 40.8	75.8 ± 33.0	60.2 ± 24.9	76.1 ± 33.5	0.51
Comfort	64.8 ± 35.5		NA	48.6 ± 42.2	76.0 ± 29.8	55.8 ± 20.6	88.3 ± 11.6	0.03
Aesthetic	65.6 ± 34.2		NA	53.2 ± 40.9	76.3 ± 26.7	70.0 ± 28.9	71.1 ± 29.7	0.28
Stability	59.7 ± 36.0		NA	53.2 ± 41.2	64.7 ± 35.5	62.0 ± 29.5	63.9 ± 32.0	0.82
Chewing ability	51.6 ± 29.2		NA	46.7 ± 29.7	52.6 ± 32.2	50.6 ± 27.9	62.1 ± 25.7	0.72
Chewing efficiency	49.9 ± 31.0		NA	41.4 ± 29.8	57.3 ± 35.0	45.0 ± 23.3	59.5 ± 29.6	0.42
Oral condition	53.4 ± 33.5		NA	51.9 ± 37.6	50.3 ± 32.7	46.2 ± 24.3	68.1 ± 32.4	0.64

α = 0.01

*NA* not applicable, *RD* removable dentures

Further analyses performed on the “Renewal RD” group showed that their mean GOHAI scores were not impacted by gender, while their mean GOHAI-Add score was significantly different according to the “age” group (below 70 years: 39.9 ± 10.7, and above 70 years: 46.1 ± 10.7) (p < 0.01). The difference was also significant for the scores of the functional and psychosocial fields for the “age” group below 70 [12.6 ± 4.8 and 15.5 ± 4.8, respectively (p < 0.05)] and for the age group above 70 [15.1 ± 3.6 and 18.7 ± 5.7, respectively (p < 0.05)]. Age and gender did not influence the MGDSI mean scores. Participants’ education level and way of life did not impact either of the scores.

### After prosthetic rehabilitation

The GOHAI mean scores (GOHAI-Add and from each field) and the mean scores from each item of the MGDSI questionnaire obtained after rehabilitation according to RD experience and each type of rehabilitation, are presented in Table 3, in association with their statistical significance. Subjects from the “Renewal RD” group still declared a poor OHRQoL (47.1 ± 10.0), while the “Control” group had an average OHRQoL (51.7 ± 7.1).

**Table 3:** Comparison of GOHAI and MGDSI scores between groups of the study after rehabilitation

Variable parameter after rehabilitation	RD experience			Types of prosthetic rehabilitation (renewal RD group only)				
	Renewal RD group n = 43	Control group n = 16	Student t test	Bi-maxillary complete RD n = 17	Uni-maxillary complete RD n = 14	Uni-maxillary partial RD n = 5	Bi-maxillary partial RD n = 7	ANOVA procedure
	Mean ± SD	Mean ± SD	p value	Mean ± SD	Mean ± SD	Mean ± SD	Mean ± SD	p value
Oral Health related quality of life assessment (GOHAI questionnaire)								
GOHAI-Add	47.1 ± 10.0	51.7 ± 7.1	0.095	42.3 ± 12.2	48.9 ± 7.9	47.8 ± 4.2	54.4 ± 4.7	0.037
Functional field	14.3 ± 3.7	17.4 ± 2.2	0.002**	12.5 ± 4.6	14.9 ± 2.6	14.6 ± 2.2	16.9 ± 2.4	0.051
Psychosocial field	20.1 ± 4.8	20.9 ± 3.8	0.547	18.1 ± 5.9	20.6 ± 3.9	20.6 ± 2.1	23.4 ± 2.1	0.074
Pain or discomfort field	12.7 ± 2.3	14.4 ± 3.1	0.021	11.7 ± 2.7	13.2 ± 2.0	12.6 ± 1.1	14.1 ± 0.9	0.068
Patient’s RD-related satisfaction assessment (MGDSI questionnaire)								
Ease of cleaning	88.4 ± 18.3	86.1 ± 23.2	0.687	86.6 ± 23.9	92.1 ± 6.1	78.2 ± 26.1	93.1 ± 8.8	0.445
General satisfaction	72.9 ± 32.1	86.6 ± 22.5	0.126	59.7 ± 41.7	82.4 ± 14.5	64.6 ± 31.2	93.4 ± 9.9	0.060
Ability to speak	81.1 ± 24.7	89.3 ± 14.5	0.219	72.7 ± 29.3	88.2 ± 12.7	76.8 ± 30.7	91.1 ± 21.7	0.228
Comfort	64.6 ± 34.1	82.8 ± 25.6	0.059	46.7 ± 37.6	72.7 ± 28.5	63.6 ± 24.4	93.7 ± 9.5	0.010** (F = 3)
Esthetic	83.7 ± 27.7	92.1 ± 15.5	0.263	69.1 ± 39.9	92.6 ± 4.7	92.6 ± 8.1	95.1 ± 7.1	0.042
Stability	68.4 ± 33.1	67.2 ± 34.3	0.900	52.2 ± 38.1	75.5 ± 28.0	74.0 ± 21.5	90.6 ± 16.7	0.040
Chewing ability	61.4 ± 29.0	79.9 ± 21.6	0.024*	55.7 ± 32.3	60.3 ± 31.0	61.2 ± 19.1	77.6 ± 19.4	0.427
Chewing efficiency	65.3 ± 28.2	72.1 ± 28.1	0.409	57.8 ± 29.5	65.2 ± 30.8	62.0 ± 22.2	86.1 ± 13.7	0.167
Oral condition	75.7 ± 26.4	81.3 ± 22.2	0.456	65.9 ± 33.6	82.9 ± 17.2	67.2 ± 24.2	91.4 ± 9.5	0.090

α = 0.01

** p < 0.01

*NA* not applicable, *RD* removable dentures

In general, for the “Renewal RD” group, the type of prosthetic rehabilitation did not significantly influence the GOHAI and MGDSI mean scores, except for the “Comfort” item. A subsequent Post ANOVA analysis (Student–Newman–Keuls) showed that the mean “Comfort” MGDSI score was significantly different for subjects that received either a bi-maxillary complete RD or a bi-maxillary partial RD rehabilitation.

Further analyses performed on the “Renewal RD” group showed gender and age had no effect on the mean GOHAI scores, or on any of the mean MGDSI scores, except for one. Indeed, the score for the item “Ease of cleaning” was significantly different according to gender (p < 0.05), with a score of 93.3 ± 13.0 for women and 81.8 ± 23.5 for men.

### Evolution of OHRQoL and RD-related satisfaction

Subjects from the “Control” group, who had no RD experience, showed an increase in their functional GOHAI field score (p < 0.01) but not for scores of the GOHAI-Add or from the other fields.

Subjects with RD renewal showed an increase in their GOHAI-Add scores (F = 15, p < 0.001), as well their psychosocial field scores (F = 25, p < 0.001) and those of the pain or discomfort field (F = 39, p < 0.001). However, the scores of the functional GOHAI field did not change. There was no impact on the variations of the GOHAI mean scores before and after prosthesis renewal due to the type of prosthetic rehabilitation. For the MGDSI, scores for the “Esthetics” (F = 39, p < 0.001), “Chewing efficiency” (F = 39, p < 0.001) and “Oral condition” (F = 39, p < 0.01) items increased after prosthesis renewal.

## Discussion

The aim of this study was to assess whether RD renewal improved patients’ OHRQoL and their RD-related satisfaction, and to assess whether improvement was influenced by the type of rehabilitation or by sociodemographic factors. For this study, the OHIP questionnaire was not chosen to assess the quality of life as no French version was validated. On the other hand, the GOHAI questionnaire was previously validated in French language by Tubert-Jeannin et al. (2003). Moreover, the GOHAI instrument contains more questions on functional aspects that the OHIP versions (Pistorius et al. 2013).

On the one hand, the results suggested that renewing RD improved patients’ OHRQoL. Indeed GOHAI-Add scores increased from 40.6 ± 10.3 before rehabilitation to 47.1 ± 10.0 after rehabilitation. Despite this increase, OHRQoL remained poor in patients with RD renewal. Furthermore, items related to the GOHAI functional field were not impacted. These results confirmed that people wearing an RD often report an affected OHRQoL (Locker and Miller 1994; McGrath and Bedi 2001; John et al. 2004). However, only few reports in literature mentioned the exact type of removable rehabilitations used. Some studies suggested that OHRQoL is degraded due to poor prosthesis quality (Inukai et al. 2008; Andrade et al. 2012). However, in the current study, RD quality was ensured by the fact that the prostheses were new and that overall treatment was supervised by a senior prosthesis practitioner. In addition endodontic, conservative and periodontic treatments were performed before prosthesis renewal. Prevention and conservative treatment remain the best way to prevent OHRQoL degradation. Several studies have shown that OHRQoL is greatly improved by the number of natural teeth present on the arches (Tubert-Jeannin et al. 2003; John et al. 2004; Hägglin et al. 2004; Pistorius et al. 2013). It has also been shown that dental care improved OHRQoL, especially in elderly people (Naito et al. 2010; İlhan et al. 2015). When rehabilitation is inevitable, RD treatment remains a valuable solution for these patients in some circumstances (financial, patient’s preferred option, etc.) (Xie et al. 2015). As shown in this study, OHRQoL remained poor after RD renewal, and the best alternative treatment would be rehabilitation with implant prosthodontics. This would greatly improve the degraded OHRQoL, as shown in several studies (Fillion et al. 2013; De Bruyn et al. 2015). It was also noticed that some patient still choose rehabilitation with RD, even when implant-supported dentures are freely offered. This option choice could be due to fear of surgical intervention (Walton and MacEntee 2005; Carlsson and Omar 2010).

On the other hand, RD renewal only had a limited impact on prosthesis satisfaction. The “general satisfaction” item remained statistically similar before and after rehabilitation (59.7 ± 35.1 vs 72.9 ± 32.1). The high variability suggested that patient’s expectations were not fully identified, probably because personality traits related to the OHRQoL were not taken into account when treatment options were decided. Takeshita et al. (2015) showed that the evaluation of patients’ personality traits would lead to a more adapted therapeutic approach. However, this approach would be difficult to implement as these require personality tests that can only be performed and analyzed by persons specialized in psychology. As an alternative, qualitative research centered on patients’ expectations could be performed to identify different patients’ profiles and determine the appropriate treatment accordingly. In contrast, other items, such as “Esthetics”, “Chewing efficiency” and “Oral condition” were significantly improved. These results, based on patient-centered/reported outcomes, tended to show that the masticatory function of patients with RD renewal was improved. In this study, however, prosthetic rehabilitation only consisted in renewing an already existing RD, limiting changes. This was confirmed by the fact that the GOHAI functional field was not improved upon RD renewal, independently of the type of prosthetic rehabilitation. Furthermore, despite the fact that patients with and without RD experience showed a similar improvement of their overall OHRQoL, patients that experienced RD for the first time were the only ones for whom OHRQoL functional features were improved. In the literature, wearing RD tends to be associated with altered mastication (Liedberg et al. 2005). Further analyses on mastication physiological parameters, including food bolus particle size, should be performed to confirm the perceived improvement of the masticatory function in patients with RD renewal.

The study of the impact of sociodemographic factors showed that, before RD renewal, age had an influence on patients’ OHRQoL. Indeed, patients over 70 years had a better OHRQoL than patients under 70, confirming the hypothesis of Hägglin et al. (2004) that older patients had a better acceptance of their condition of life. After RD renewal, age was no longer an influencing factor, as OHRQoL is similar between both age groups. The ability of older patients to adapt to a new RD may be diminished. Moreover, according to the literature, gender did not have an impact on OHRQoL at any stage (Tubert-Jeannin et al. 2003; John et al. 2004). In accordance with a study concerning patients with bi-maxillary complete RD (Turker et al. 2009), neither age nor gender had an impact on RD-related patient satisfaction, except for one item, “Ease of cleaning”. Indeed, after RD renewal, prosthesis cleaning is easier for women than for men. This aspect of cleaning behavior should be taken into consideration for RD upkeep, as it was also done for tooth brushing (Wiener et al. 2012).

Patients’ OHRQoL was not impacted by the type of rehabilitation before or after RD renewal. A similar result was obtained for the patients’ RD-related satisfaction. However, on the present study, patients that had a bi-maxillary complete RD renewal experienced the worst comfort sensation while patients that had a bi-maxillary partial RD renewal experienced the best comfort of all the other rehabilitation types. Other authors reported a better OHRQoL for patients wearing bi-maxillary complete RD (Yen et al. 2015). This was explained by the fact that people with partial dentures tended to compare their dentures with their remaining natural teeth. On the contrary, one could also say that people wearing complete dentures had forgotten the feeling of having teeth, and therefore had more expectations while wearing complete dentures. These persons experienced a worst comfort sensation that influenced their reported OHRQoL. For patients with a complete denture it would be necessary to recommend an implant prosthodontic rehabilitation according to the McGill consensus (Feine et al. 2002) and the York statement (Thomason et al. 2009), in which the mandibular prosthesis is retained by two implants. In France, this protocol is difficult to implement due to financial issues and the lack of insurance coverage for dental implants, therefore an adapted public health program should be put in place in order to provide this therapeutic approach for patients.

Caution is required regarding the results from the OHRQoL analysis. Indeed, although the GOHAI questionnaire has been validated for France (Tubert-Jeannin et al. 2003), the results obtained are closely related to the population studied. Therefore, these results cannot be standardized for another culture, or treatment (İlhan et al. 2015).

## Conclusions

Within the limitation of the present study, it can be concluded that renewing RD only moderately improved OHRQoL and RD-related satisfaction, regardless of age, gender or type of rehabilitation. Other aspects such as the analysis of physiological parameters and qualitative research on patients’ expectations should be investigated.

## Abbreviations

RD: removable dentures
OHRQoL: oral health related quality of life
GOHAI: Geriatric Oral Health Assessment Index
MGDSI: McGill Denture Satisfaction Instrument
